# Enhancing patient-provider encounters when proposing SDF therapy by utilizing a qualitative analysis of parental feedback

**DOI:** 10.3389/froh.2024.1421157

**Published:** 2024-09-19

**Authors:** Yasmi O. Crystal, Jenny Hiyeji Jang, Victoria H. Raveis

**Affiliations:** ^1^ Department of Pediatric Dentistry, New York University College of Dentistry, New York, NY, United States; ^2^ Private Practitioner, Fort Lee, NJ, United States; ^3^ Psychosocial Research Unit on Health, Aging and the Community (PRUHAC), Department of Cariology and Comprehensive Care, New York University College of Dentistry, New York, NY, United States

**Keywords:** silver diamine fluoride (SDF), caries arrest, minimally invasive therapies, parental perceptions and satisfaction, patient-centered care, SDF informed consent, Precaution-Adoption Process Model

## Abstract

**Purpose:**

Silver Diamine Fluoride (SDF) is a minimally invasive option for caries arrest, part of a paradigm shift in the management of pediatric dental caries. The perspective of parents regarding the long-term pros and cons of this therapy should be understood in order to achieve optimal patient-centered care.

**Methods:**

This study used Constant comparative analysis as an analytic approach, applying the Precaution-Adoption Process Model (PAPM) as the Grounded Theory framework in the qualitative analysis of 30 parental unformatted, spontaneous comments collected at the end of a questionnaire to evaluate their satisfaction with treatment provided at a University Clinic.

**Results:**

Our analysis provided important insights about the factors that influenced the parents’ decision to act and have their child receive SDF therapy, their perception of the outcomes, the necessary follow-ups after the therapy, and what impacted on their overall satisfaction with the completed procedure. Both positive and negative themes were identified. The positive themes point to SDF treatment's ease of application and addressing the immediate treatment needs on children with limited cooperation. The negative themes identified the adverse consequences of SDF treatment, specifically, the duration and appearance of the cosmetic consequences, as well as the parents’ misunderstandings and incorrect expectations of the long-term sustainability of the treatment, which in many instances requires further interventions. It was also evident from the parents’ comments that they needed additional educational guidance on other aspects of the treatment, such as the necessity for clinical follow-ups, information that impacted parents’ overall satisfaction with the treatment their child received.

**Conclusion:**

Our results highlight the need to discuss the short and long term benefits of the treatment, as well as, its short and long-term limitations. Specifically, while it is important to discuss immediate outcomes and consequences, such as the ease of treatment and the resultant staining, to ensure that parental consent for the treatment is truly well-informed, it is also important to prepare parents, when this procedure is initially proposed, of the likely need for additional oral care interventions in the future.

## Introduction

Treatment of dental caries has traditionally been focused on restoring cavities and, in the last few decades, these restorations have mainly been comprised of composite resins, compomers, glass ionomers and stainless-steel crowns (1). For these traditional restorative treatments to be successfully completed, patient cooperation is required, as most of the procedures are technique-sensitive, meaning they require isolation from saliva, ideally using rubber dam. The use of local anesthesia is necessary not only for rubber dam placement, but also for caries removal and restoration placement procedures, especially when caries is near the pulp and the tooth requires pulp therapy. Many children are unable to achieve the levels of cooperation needed to complete these procedures successfully, leading to the need for advanced forms of behavior guidance, such as sedation and general anesthesia, which increase the risk, cost, and potential barriers to receiving the necessary treatment (2). Parents are often distressed by the scenario of confronting their children's dental needs and the children's inability to cooperate to get the treatment done.

For these reasons, the advent of alternative approaches for caries management like silver diamine fluoride (SDF), intermediate therapeutic restorations (ITR), atraumatic restorative treatment (ART) and performed crowns placed with the Hall technique or with modifications of this technique (Hall style crowns) have gained popularity in pediatric dentistry (3–6). With the advent of better materials, the increased longevity and potential success of these procedures present the opportunity for a paradigm shift in the management of dental caries in pediatric populations. For the treating clinician, being able to control the caries progression of early and moderate lesions, even as a temporary measure, means that the tooth can be preserved, perhaps delaying or avoiding the need of more involved treatment or tooth extractions.

There is ample evidence in the literature of the efficacy of SDF to arrest dentin caries lesions in primary teeth when applied twice a year, with most studies reporting results of caries arrest over a 24-month period. Arrest rates range from 40% to 90%, depending on many variables, such as position and extent of the cavity, area of the mouth treated, ability to keep the cavity free of plaque, and frequent exposure to a cariogenic diet (7–9). Its use is supported by national and international guidelines of recognized organizations like the American Academy of Pediatric Dentistry (AAPD), the American Dental Association, the International Association of Pediatric Dentistry, and others. AAPD's recommendations are to use SDF as part of a comprehensive caries management plan, where: treated teeth can be followed-up and assessed for sustained caries arrest, programs are established to control variables that will reduce the risk factors associated with diet and home care, and to offer alternative solutions for tooth restoration whenever possible and necessary (10). The program directors of most pediatric dentistry programs in the US report using SDF as an interim treatment, and the later placement of a glass ionomer or other restorative material depends on multiple factors, such as the life expectancy of the tooth, patient behavior, overall caries risk, treatment setting, goal of SDF placement and parental preferences (3). Many studies cite the dark staining, characteristic of lesion arrest, as a major barrier in the utilization of SDF, as parents often do not consent to its use in visible areas due to esthetic concerns (11, 12).

If we are to support SDF as a viable alternative option, part of a paradigm shift in the management of pediatric dental caries, it is of the utmost importance to understand the viewpoints and concerns of parents. Their perspectives regarding the long-term pros and cons of this therapy after having experienced it on their children, should be studied in order to achieve optimal patient-centered care. In a previous study, where we followed up SDF cases from our university pediatric specialty clinic, we found that at least half of the treated teeth received further treatment. However, parental satisfaction with the SDF treatment option remained high, due to the ease of application and desensitizing effects. Our statistical analysis confirmed that in instances where parents expressed dissatisfaction, their negative rating was related to the staining of anterior teeth, the need for further treatment, and a lesser understanding of side effects. These quantitative results were reported in a previous publication (13). As part of that investigation, parents were also invited to share their viewpoints and concerns as open, unformatted opinion statements, regarding the pros and cons of the SDF treatment that their child received as part of the comprehensive, patient-centered care that was delivered at this university pediatric dentistry clinic. The aim of this study was to perform a qualitative analysis of the comments provided by parents to understand their perspectives on the long-term effects of SDF treatment.

## Methods

This study was reviewed and approved by the New York University Institutional Review Board (NYUIRB Study #S19-01393). Parents whose children received SDF treatment from February 1, 2019 to February 29, 2021, at our university Pediatric Dentistry clinic, were contacted by telephone to participate in the study. No exclusions were applied. Parents were presented with an IRB approved consent form, and those who verbally agreed to participate, answered questions from a data collection instrument that was designed to obtain information about the ease of treatment, their child's experience with SDF treatment, their recollection of the outcome of the tooth/teeth treated, their understanding of the benefits and side effects of the treatment, and their level of overall satisfaction. This questionnaire, tested for clarity, precision and timing in English and Spanish, was administered in English by a pediatric dental resident and in Spanish by a Spanish speaking 4th year dental student. They were both trained and calibrated to deliver the verbal questionnaire, and to collect the data. Telephone interviews were conducted between July and November 2022. Concurrently, data was extracted from their child's dental records including gender, age, household phone number, general health status, dental insurance, tooth/teeth treated with SDF and the outcome of the treated tooth, if available. More details of the questionnaire and results from the quantitative analysis of the data obtained have been published recently (13). At the end of the questionnaire, parents were given the opportunity to comment or expand on their views of the treatment or the overall study. Many parents shared their point of view, providing valuable comments about significant issues that are the object of analysis in this study.

The parents’ comments were audiotaped, transcribed verbatim, and stored in NYU Langone Health REDCap (Research Electronic Data Capture), a web-based application developed by Vanderbilt University to capture health data. It is Health Insurance Portability and Accountability Act (HIPAA) compliant and highly secure (14). The parents’ spontaneous comments were then subjected to qualitative analysis using grounded theory approaches. Constant comparative analysis (15) was the qualitative analytic approach used to identify both the positive and negative themes that emerged when focusing specifically on a parent's comments regarding his/her child's SDF treatment. In applying this analytic approach, each author (medical sociologist, experienced pediatric dentist and pediatric dentistry resident who were not the providers of the SDF therapy) individually reviewed each of the comments and linked it to a theme. Then theme blocks were examined, discussed and streamlined into final categories by the three authors until a consensus was reached. Themes were continually re-examined, expanded upon and revised, as necessary, each time a parent's comment was added to it. This was done with the three authors over several sessions to insure an adequate verification process. Once themes were finalized, the Precaution-Adoption Process Model was used as a framework (15) to identify the factors that influenced parents’ decision to act and have their child receive SDF therapy, their perception of the necessary follow-ups after the therapy, and what impacted on their overall satisfaction with the completed procedure.

## Results

Of the 79 parents who participated in the original study answering the full questionnaire, 30 parents also shared significant comments about the SDF treatment their child received, enabling further insight about their perspectives. Parents’ unedited comments are included in Table 1, highlighting those parents whose children have SHCN. From the constant comparative analysis of these comments, several major themes emerged: *Esthetic concerns, Tooth location, Child's age, Addressing parental short- and long-term treatment outcome expectations, Children with special needs, and Merits of SDF as an alternative treatment in special circumstances*.

**Table 1:** Unedited comments from the 30 parents who choose to elaborate after the questionnaire.

Parent #	Comments from parents
	Child with Special Healthcare Needs^a^
	Parent or child bothered by discoloration^b^
	Would not do SDF again^c^
1	I understand that the advantages were that it would stop the cavities from getting worse, but it was not clear about the side effects
2	I really liked the treatment but the color is not very pleasing. I like NYU and everyone is nice there but I couldn't go to the follow up appointments because it's too far
3	I was very happy with this treatment. I want to go back to NYU but they don't accept my insurance
4	I was happy they were able to save it for 3 years because those teeth weren't going to last. My daughter is a disabled child and is non-verbal, so it was really difficult to get any treatment done on her
5	I didn't want to take out the teeth, but they ended up taking it out
6	It didn't bother me at all. It was the only solution for my autistic son
7	It's easy treatment for a child but I'm not that happy because she still had a hole
8	My son is very young so he wasn't bothered or didn't seem to care about the staining
9	The resident and supervisor explained the treatment and procedure very well. I think my son isn't bothered because he wears a mask to school every day. I would choose this treatment because its less pain and its quick
10	My child wasn't bothered about it when she first got it but once she went to school and got a little older the staining started bothering her
11	It was challenging because my child is so scared and strong. This treatment was good for my children because they are scared
12	No other complaints except I don't like how the teeth turned black
13	I'm satisfied because the silver cap is still there, but I wouldn't want my child's teeth to show black
14	I'm satisfied that it can help with the cavities
15	It was a good medicine. The tooth turned black a little bit so it didn't bother me or my child much
16	I'm very satisfied with the treatment it removed the pain my child had
17	They explained to me that it was a new treatment that would be easy to be done and would stop the progression of the cavity so I was happy to do it
18	Good treatment but the wait time at NYU is too long
19	I liked the treatment, they gave us very good attention and they treated my girl very well
20	I didn't notice the staining but there's silver cap on it now. It didn't bother me
21	I thought it was a good and easy option because my child is special needs and is very active
22	I liked the treatment, it did not change the color of the tooth much and they treated my daughter very well and I am satisfied with the treatment
23	I'm not sure if the treatment really worked or not because she was in the transition stage and many teeth have been falling out in the past 1–2 years
24	It was in the back so me and my child wasn't conscious of it. If the cavity is in the back, I would recommend it because you can't see it. My daughter cares a lot about her look, so I wouldn't want that in the front teeth
25	It didn't really bother my child most of the time but when people asked him about his black teeth he would ask me why it turned black
26	My child has no more teeth problems so I'm happy with the treatment
27	My son always vomits and is very scared of the dentist, so he needed this medicine to stop the cavity from getting bigger
28	It's in the back so it doesn't bother me or my child. I think this is a really good treatment option
29	It has a tiny small black dot that didn't get bigger and it didn't get worse so it was a good treatment. The disadvantage is that it turns black so I would recommend it only for baby teeth and if it's for back teeth but I wouldn't recommend it for front teeth
30	The only thing is that they couldn't put the treatment on the other teeth because they said that the cavity was too big and we had to wait so long for him to get treatment under GA

^a^ SHCN was determined from the medical history data provided by the parent.

^b,c^ As reported by the parent in the questionnaire: Crystal et al. (13).

### Esthetic concerns

Esthetic concerns associated with the black staining were the most common source of dissatisfaction with this treatment option. As one parent noted: “I really liked the treatment, but the color is not very pleasing.” Other parents provided similar comments, such as: “No other complaints except I don't like how the teeth turned black”. Several parents shared that they had not fully understood the treatment consequences. They stated that they were unprepared for the tooth's appearance—that the treated area would turn black. They also did not realize that this discoloration of the tooth would be permanent. Other parental comments reflected the child's awareness of the discoloration: “It didn't really bother my child most of the time but when people asked him about his black teeth he would ask me why it turned black.”

### Tooth location

The location of the tooth was a factor in whether any esthetic concerns due to tooth staining adversely impacted parental satisfaction with the treatment. As one mother explained: “It was in the back so me and my child wasn't conscious of it. If the cavity is in the back, I would recommend it because you can't see it. My daughter cares a lot about her looks, so I wouldn't want that in the front teeth.” Another mother shared: “It has a tiny small black dot that didn't get bigger and it didn't get worse, so it was a good treatment. The disadvantage is that it turns black, so I would recommend it only for baby teeth and if it's for back teeth, but I wouldn't recommend it for front teeth”.

### Child's age

The age of the child also impacted whether parents viewed the staining as problematic. Since the tooth being treated may remain present for a few years before the permanent tooth erupts, pre-school children might become upset with their appearance once they start school. Such an occurrence was described by one mother: “My child wasn't bothered by it (the staining) when she first got it, but once she got a little older and went to school, the staining started bothering her.” Other parents also stated that it was not an issue yet because of the child's early age. As one parent shared: “My son is very young, so he wasn't bothered or didn't seem to care about the staining. Other parents’ comments noted that an advantage of using this treatment on older children's teeth is that the tooth will soon be exfoliated. As one parent reflected: “I'm not sure if the treatment really worked or not because she was in the transition stage and many teeth have been falling out in the past 1–2 years”.

### Addressing parental short- and long-term treatment outcome expectations

It is essential that the child's parents have a clear understanding of the treatment's short- and long-term benefits, as well as, the negative consequence (permanent staining), relative to the standard traditional dental care. If this understanding of the long-term outcome is not achieved, the treatment is likely to generate parental dissatisfaction with their child's care and/or regrets regarding their care-management decisions. As one parent explained: “I understand that the advantages were that it would stop the cavities from getting worse, but it was not clear about the side effects [permanent staining].” Another parent complained that she was not told and did not realize that the hole made by the cavity would not be filled as part of the treatment procedure. As she commented: “It's an easy treatment for a child but I'm not that happy because she still had a hole.” Another parent shared that she was not aware that while the treatment is highly effective, it does not work 100% of the time. As she stated: “I didn't want to take out the teeth, but they ended up taking it out”.

### Children with special needs

Parents of children with special needs were generally enthusiastic about the minimally-invasive treatment option SDF offered, noting that the treatment was well-tolerated by their child and regarded the black staining as a minor concern when weighed against the benefits of being able to utilize a non-invasive, brief procedure to address cavities in their child's primary teeth. As one mother explained: “I was happy they were able to save it for three years because those teeth weren't going to last. My daughter is a disabled child and is non-verbal, so it was really difficult to get any treatment done on her.” Another parent commented further: “It didn't bother me at all. It was the only solution for my autistic son.” Additionally, parents shared that they found this treatment option to be beneficial for their children who suffer from dental anxiety. As one parent related: “My son always vomits and is very scared of the dentist, so he needed this medicine [SDF] to stop the cavity from getting bigger”.

### Merits of SDF as an alternative treatment in special circumstances

Several circumstances, that were less commonly mentioned, merit consideration when recommending SDF to parents as an alternative dental treatment. For example, the advantages of SDF as a dentin desensitizer. As one parent shared: “I'm very satisfied with the treatment, it removed the pain my child had”. Another less commonly mentioned benefit that some parents alluded to in their comments, is the ease of treatment. As one parent explained: “I think my son isn't bothered because he wears a mask to school every day. I would choose this treatment because it's less pain and it's quick”. Other parental comments pointed out that although the treatment may still be challenging for children with poor cooperation, it was the better choice as it was faster to complete than other conventional treatments. This sentiment is reflected in the following parent's statement: “This treatment was good for my children because they are scared”.

Parents’ negative comments regarding the staining revealed a lack of prior understanding related to the side-effects of the treatment, specifically that the procedure permanently stains the treated tooth black, and this staining does not fade or disappear over time. The parents’ reports of the adverse psychosocial consequences that this black staining had on their child's appearance and the child's subsequent loss of self-esteem in those instances when the treated tooth was a visible front tooth underscores the importance of a careful dialogue with the parent when discussing treatment expectations and potential short- and long-term adverse psychosocial outcomes for their child. The parents’ reports also revealed that some parents expected the cavity hole to be filled as part of the SDF treatment. This is another important area of misunderstanding about the procedure's outcome that should be carefully addressed when this procedure is proposed.

Through the Precaution-Adoption Process Model framework, we can see how parents’ comments reflect how they have been informed about the issue of their child's dental problems aggravated by their medical condition or their limited cooperation for treatment. Comments also depict how parents were presented with the action of choosing the SDF treatment and taken action to go through the procedure with its pros and cons. Maintenance of the action was reflected in the comments that reflect on what happened after the treatment was delivered and how will that influence their future choices.

## Discussion

The Precaution-Adoption Process Model was used to guide the analysis of the parents’ comments regarding their decision to consent to the use of SDF therapy. The Precaution-Adoption Process Model (15) identifies the stages that individuals move through along the path from a lack of awareness about a health issue, through awareness of the issue, deciding whether to take action on this issue, taking action, and maintenance of the action. The objective of utilizing this framework was to guide the qualitative analysis with an established grounded therapy approach to provide insights about the factors that influenced the parents’ decision to act and have their child receive SDF therapy, their perception of the necessary follow-ups after the therapy, and what impacted on their overall satisfaction with the completed procedure.

### Informed of the issue, presented with the action and taking action

When presented with the SDF therapy option, some parents shared that they decided upon SDF therapy out of concern for how their child would cooperate or behave if the lesion received traditional treatment, which is supported in the literature (16). Comments from parents with anxious or other special health care needs children, clearly expressed the value of the ease of the treatment, which is consistent with findings from other studies (17, 18).

Other parents were concerned about the esthetic impact of the treatment outcome, which depending on the size and/or location (anterior or posterior) of the lesion, would adversely impact their overall satisfaction of the SDF therapy. This is consistent with results from previous studies (19). Communication misunderstandings or incomplete communication regarding the SDF therapy procedure emerged as a primary source of parental dissatisfaction following the procedure. The need to ensure that parents fully understand not only the clinical benefits of the procedure, as well as, its side effects before performing the procedure, was apparent in the parents’ comments. It is important to note that all of the parents had originally signed an informed consent for SDF treatment that included photographs of teeth before and after the treatment. Despite this, our findings reveal that for some parents, the information given during that process might have been insufficient for them to understand all the implications. Use of printed information and consent forms with photographs and a concise but accurate written description of long-term side effects, stressing expected outcomes and treatment goals may be helpful. Allowing parents to re-read the information at home at a more leisurely pace may allow them to fully understand potential outcomes.

### Maintenance of the action

The location of the tooth was an important factor in parental satisfaction with the procedure. The age and functional status of the child were factors that impacted parental satisfaction with the outcome of the procedure. A very young child, or a special needs patient may benefit from a brief, less invasive procedures. Contributing to the parents’ dissatisfaction, which some parents’ expressed with the tooth staining procedure, was the appearance of a hole in the tooth or the tooth ultimately needing to be pulled. The fact that this procedure was an interim solution was beneficial in those situations where the tooth would be falling out soon or when it was applied to a non-primary tooth. Some of the comments pointed out logistical barriers that hampered continued receipt of the treatment, and treatment that parents wanted to continue, as the following comment illustrates: “I was very happy with this treatment. I want to go back to the clinic, but they don't accept my insurance”.

As suggested by the precaution adaptation process, the parents’ comments indicated that maintaining good communication with the parent when discussing the clinical benefits, as well as the consequences, was valued. These findings strongly support the importance of ensuring that parents fully understand the treatment's side effects, and its clinical benefits. Indeed, most parents offered positive comments, sharing that SDF therapy was an easy, non-invasive, quick and pain-free experience for their child. Many of the parents, who positively endorsed SDF treatment, shared that they were content with the outcome, even though this option only provided a temporary solution, as their special needs child benefited from the brief, less invasive procedure,

Furthermore, the parents’ comments also indicated that consideration needs to be given not only to the clinical condition of the tooth, but also to the location of the tooth and whether it is a primary or permanent tooth. Overall, the findings from this investigation indicate that most parents, particularly those whose children had special healthcare needs or exhibited poor cooperation to receive traditional restorative treatment, were satisfied with SDF therapy. These findings support the clinical utility of offering this option to patients when the delivery traditional treatment would be challenging, and has been reported in other studies (20).

It merits noting that the negative comments parents shared regarding SDF therapy were related to a misunderstanding and/or poor explanation of what SDF therapy entails and the staining it caused. Parents expressed dissatisfaction when further treatment was required. They were also concerned about the social and psychological impacts the staining had on their child. The use of a written form for parents to take home with concise, relevant information about the treatment may overcome any misunderstandings that can occur when parents are making decisions under time pressure, and with any variability in different providers’ explanations.

Through this study, we were able to understand how to improve the patient-provider encounter. The most crucial aspect in improving the parents’ satisfaction is ensuring the parents have an adequate understanding of the goal of SDF therapy which is to stabilize dental disease until the patient can complete definitive treatment, whether it is when they become more cooperative to sit for treatment, or until they can be seen for treatment under general anesthesia (21). In cases where the treated tooth is near exfoliation, it may not need future conventional treatment. However, SDF has to be re-applied every six months to ensure continued arrest. After SDF therapy, the final treatment varies on different factors that can only be evaluated with periodic monitoring of the tooth, which can result in restorations such as fillings, crowns, or extractions.

It is also important that parents and providers share a mutual understanding of the success of SDF therapy in which the goal is to arrest disease and prevent further deterioration of the lesion. Though SDF therapy provides a fix to the problem, at that moment, it is important to emphasize the interim/temporary nature of this treatment and thoroughly explain to the parent both, the short-term and long-term expectations for this therapy. To improve understanding, pictures are encouraged for visual reference of the side effects of SDF therapy. With a deeper understanding of the goals, effects, and impacts of this treatment, parents will be more accepting of this therapy as a whole.

One limitation of our study is that although parents were asked to freely express their feelings about the treatment and their experience with it, the telephone interviews could have missed capturing the non-verbal messages that an in-person interview could have offered. Another potential limitation is that this group of patients may have had an inherent bias since they had already been presented with an informed consent and had agreed to receive the treatment. Parents completely opposed to any aspect of SDF would have chosen other options like waiting for general anesthesia, no treatment or emergency extractions. However, one of the strengths of our study is that children in the study had received the treatment at least 1 year before the interview, so parents had experienced dealing with the benefits and limitations of the therapy.

Another aspect that could not be explored based on our data, is the economic aspect of the therapy. Parents that attend our clinic typically have state insurance that covers all treatment their child requires and therefore, their comments do not reflect any economic aspects of the advantages of SDF being a more affordable option for families with limited resources. This important aspect merits further investigation in a setting where the choice of therapy is influenced by economic limitations.

When treating pediatric patients, providers need to construct treatment plans tailored to each individual that is dependent on their age, cooperativity, health, and extent/amount of dental disease. SDF therapy can eliminate treatment barriers. It is a great tool to present as an alternative treatment option to traditional dental treatment, so it is important to help parents in all the areas to make that will allow them to make the right choice for their child. As some patients mentioned financial limitations for attending our clinic or for choosing the treatment options, it would be important for third party payers to acknowledge the interim nature of the SDF treatment when considering coverage for its need of frequent periodic surveyance for re-application, or for future interventions that will be required on treated teeth.

## Conclusion

An examination of the parental comments has supported the importance of assessing parents’ understanding of the procedure and its short and long-term impact. This analysis has also provided insights on the factors that merit discussion when this procedure is proposed to the parents to achieve a truly well-informed consent for the treatment. Both positive and negative themes were identified in this analysis. The positive themes supported SDF treatment's ease of use and immediate addressing the clinical problem. The negative themes identified the adverse consequences of SDF treatment, specifically, the duration and appearance of the cosmetic consequences of the treatment, as well as the parents’ misunderstandings and incorrect expectations of the likely need for further treatment. These findings support the clinical importance of ensuring that parents fully understand both the benefits of SDF treatment, as well as the negative consequences (e.g., permanent staining and interim nature) of the procedure when treatment options for their child are discussed. Focusing on the commonalities and differences in the parents’ comments has provided a breath of insights about the SDF treatment experience that inform clinical practice to achieve the goal of delivering patient-centered care.

## Data Availability

The original contributions presented in the study are included in the article/Supplementary Material, further inquiries can be directed to the corresponding author.
